# CEA: Combination-based gene set functional enrichment analysis

**DOI:** 10.1038/s41598-018-31396-4

**Published:** 2018-08-30

**Authors:** Duanchen Sun, Yinliang Liu, Xiang-Sun Zhang, Ling-Yun Wu

**Affiliations:** 1 IAM, MADIS, NCMIS, Academy of Mathematics and Systems Science, Chinese Academy of Sciences, Beijing, 100190 China; 2 School of Mathematical Sciences, University of Chinese Academy of Sciences, Beijing, 100049 China

## Abstract

Functional enrichment analysis is a fundamental and challenging task in bioinformatics. Most of the current enrichment analysis approaches individually evaluate functional terms and often output a list of enriched terms with high similarity and redundancy, which makes it difficult for downstream studies to extract the underlying biological interpretation. In this paper, we proposed a novel framework to assess the performance of combination-based enrichment analysis. Using this framework, we formulated the enrichment analysis as a multi-objective combinatorial optimization problem and developed the CEA (Combination-based Enrichment Analysis) method. CEA provides the whole landscape of term combinations; therefore, it is a good benchmark for evaluating the current state-of-the-art combination-based functional enrichment methods in a comprehensive manner. We tested the effectiveness of CEA on four published microarray datasets. Enriched functional terms identified by CEA not only involve crucial biological processes of related diseases, but also have much less redundancy and can serve as a preferable representation for the enriched terms found by traditional single-term-based methods. CEA has been implemented in the R package CopTea and is available at http://github.com/wulingyun/CopTea/.

## Introduction

Functional enrichment analysis is a fundamental and challenging task in bioinformatics. The arrival of high-throughput technologies, such as next generation sequencing, single-cell sequencing^1^, and Hi-C^2^, has produced huge amounts of experimental data in the past few decades. Interpreting and analyzing the functions of key genes identified from these high-throughput experiments in a systematic level is extremely important in the post-genomics era. A common step in the downstream analysis of high-throughput experiments^3^ is gene set functional enrichment analysis, which aims to investigate the functional associations between a gene list of interest and the specific reference gene sets annotated with common functions. The important genes (aka active genes) identified from biological experiments are often represented by the involved functions (e.g. in biological processes, cellular components, molecular functions and signal pathways), and a systematic panorama of phenotypes in experiments is derived using the back-end annotation databases, for example, Gene Ontology (GO)^4,5^, KEGG^6^, OMIM^7^ and MSigDB^8,9^.

The functional terms often exhibit high similarity and redundancy within and between annotation databases. For example, GO terms overlap with their descendants in the hierarchical structure of GO annotations. The KEGG pathways may largely overlap with the GO terms of similar functions. Traditional enrichment analysis methods that individually evaluate the functional terms assign close enrichment scores to similar terms and output a long list of enriched terms, which contain similar and redundant terms and make it difficult for biologists to analyze and reveal the underlying interpretations. During the past decade, a number of computational models were proposed to make up this drawback^3,10^. Among these methods, modular enrichment analysis methods, which consider the relationships between terms and return the most enriched term module, have received increasing attentions^3^, e.g., DAVID^11,12^, GOMA^13^, MGSA^14^, GenGO^15^, MCOA^16^, MFA^17,18^ and SLPR^19^.

Two very different strategies that evaluate sets of terms at a time have been proposed to overcome the weaknesses of single-term-based approaches. The first approach groups similar terms into a cluster and evaluates the enrichment of functional clusters^11–13^, instead of each individual term within the clusters. The most famous cluster-based method is DAVID^11,12^. The second method aims to identify the most significantly-enriched combination of terms with complementary effects^14–19^. In the term combination, each single term is responsible for explaining a distinct subset of active genes. One single term may be not enriched in the active gene list, but the complementary effects between the terms ensure the overall performance of the combination. GenGO^15^ and SLPR^19^ are two representatives of the combination-based method.

The cluster-based approaches are fast, and their results are closely related with the inherent term clustering algorithm; however, the identified term clusters are usually evaluated separately and the relationships between clusters are not taken into consideration. While these approaches can reduce redundancy to some extent, they often generate somewhat rough results. There exists a difficult trade-off between the redundancy and the roughness. In contrast, combination-based approaches place the emphasis on the relationships between different terms and the identified terms are often distinct from each other. An enriched term combination is assumed to fully explain almost all active genes, and often consists of representative terms, each of which can individually explain a subset of active genes. Usually, combination-based approaches have more complicated models, which are built based upon different hypotheses of the observed active gene list. Both cluster-based and combination-based approaches can greatly eliminate the redundancy in the output of enrichment analysis. The combination-based methods can further help researchers to investigate the potential associations and interactions between enriched functions. Therefore, we will focus on the combination-based enrichment analysis methods in this paper.

Most existing combination-based enrichment analysis methods have a principal hypothesis about the active gene list. For example, MGSA, GenGO, MCOA and MFA assume that the active gene list is explainable via gene set activation by using a generative model and SLPR assumes that the activity of multiple gene sets has an additive impact on the gene-level statistics. Using simulated data with a known ground truth, the performance of different methods can be assessed using measures such as accuracy, receiver operating characteristic (ROC) curves, and precision-recall curves. However, the evaluation based on simulation might be strongly biased toward the methods with similar hypothesis as the simulation^19^. On the other hand, it is difficult to evaluate and compare the results of different combination-based methods for real data. This can confuse end users and make choosing an appropriate model and related parameters difficult^20^. Therefore, biologists need a unified approach to serve as a benchmark for evaluating and comparing these methods in a comprehensive perspective.

In this work, we proposed a novel statistical framework for evaluating term combinations, in which each term combination is regarded as a pseudo composite term and assessed by the Fisher’s exact test^21^. Unlike single-term-based methods, it is computationally intractable to enumerate and evaluate all possible term combinations due to the combinatorial explosion, i.e., the number of possible term combinations increases exponentially. To address this issue, we formulated the problem of identifying the most enriched term combination into a multi-objective combinatorial optimization problem. Based on this framework, we developed a novel method named CEA (Combination-based Enrichment Analysis) to perform the enrichment analysis. The advantages of CEA can be briefly summarized as follows. First, CEA not only outputs the most enriched term combination, but also clearly shows the distribution of candidate term combinations. This landscape of term combinations is a good benchmark for us to evaluate the existing combination-based functional enrichment analysis methods in a comprehensive perspective. Second, CEA does not require an inherent hypothesis about the generation of active gene lists. By using the multi-objective optimization framework originating from the Fisher’s exact test, CEA can obtain enriched term combinations with comparable performance with other hypothesis-driven approaches. Third, the output criteria of CEA, such as the size of the final output, the p-value cutoff, and the coverage, are very flexible, which allows users to customize based on specific research aims and requirements. Last but not least, CEA is a universal tool that can be applied on any species and any functional annotations in real applications.

## Results

### GO terms identified by CEA involve crucial biological processes

To test the effectiveness of CEA, a novel combination-based gene set functional enrichment analysis method, we evaluated its performance on four real microarray datasets of complex human diseases. The procedure for generating the active gene list from each dataset can be found in Materials and Methods.

**Figure 1:**
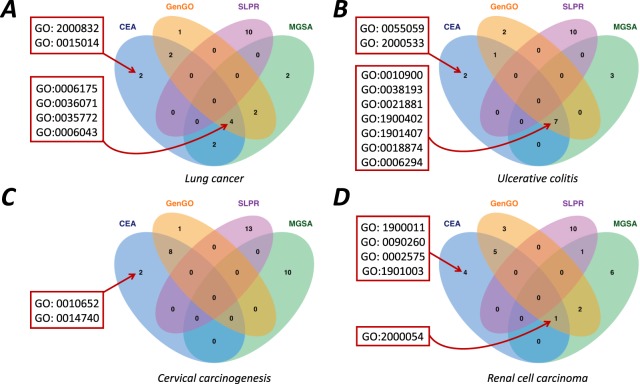
Relationships of the identified terms by each method on four-real datasets. The terms that were particularly identified by CEA and the most frequent terms (identified by at least 3 methods) were shown in red box.

In this section, we reported the most enriched 10 GO terms identified by CEA and compared them with the results of GenGO, MGSA and SLPR (Fig. 1). The full lists of enriched terms identified by each method can be found in Tables 1–4 and Supplementary Materials (Tables S2–S13). The terms identified by CEA have large overlap with term sets identified by other methods and the specific identified terms are also not irrelevant terms, which are biologically meaningful as shown below.

**Table 1:** The enrichment analysis result of CEA on lung cancer dataset.

GO ID	Description	Rank	p-values
GO: 0006175	dATP biosynthetic process	10	5.54e-3
GO: 0036071	N-glycan fucosylation	11	5.54e-3
GO: 0035772	interleukin-13-mediated signaling pathway	12	5.54e-3
GO: 0060967	negative regulation of gene silencing by RNA	15	5.54e-3
GO: 0060784	regulation of cell proliferation involved in tissue homeostasis	17	5.54e-3
GO: 0006043	glucosamine catabolic process	18	5.54e-3
**GO**: **2000832**	negative regulation of steroid hormone secretion	30	1.11e-2
GO: 0072318	clathrin coat disassembly	34	1.11e-2
GO: 0021648	vestibulocochlear nerve morphogenesis	37	1.11e-2
**GO: 0015014**	heparan sulfate proteoglycan biosynthetic process, polysaccharide chain biosynthetic process	56	1.65e-2

The Fisher’s exact test p-values and ranks for each single GO term were also listed. The p-value of the term combination was shown at the bottom of the table. The boldfaces were the GO terms identified only by CEA, compared with GenGO, MGSA and SLPR.

Term combination p = 4.187e-20.

**Table 2:** The enrichment analysis result of CEA on ulcerative colitis dataset.

GO ID	Description	Rank	p-values
GO: 0010900	negative regulation of phosphatidylcholine catabolic process	47	3.83e-3
**GO**: **2000533**	negative regulation of renal albumin absorption	51	3.83e-3
GO: 0038193	thromboxane A2 signaling pathway	52	3.83e-3
GO: 0021881	Wnt-activated signaling pathway involved in forebrain neuron fate commitment	53	3.83e-3
GO: 1900402	regulation of carbohydrate metabolic process by regulation of transcription from RNA polymerase II promoter	54	3.83e-3
GO: 1901407	regulation of phosphorylation of RNA polymerase II C-terminal domain	55	3.83e-3
GO: 0018874	benzoate metabolic process	56	3.83e-3
GO: 0006294	nucleotide-excision repair, preincision complex assembly	57	3.83e-3
GO: 0007439	ectodermal digestive tract development	92	7.65e-3
**GO**: **0055059**	asymmetric neuroblast division	131	1.15e-2

The annotations are the same as in Table 1.

Term combination p = 8.216e-23.

**Table 3:** The enrichment analysis result of CEA on cervical carcinogenesis dataset.

GO ID	Description	Rank	p-values
GO: 0086042	cardiac muscle cell-cardiac muscle cell adhesion	139	6.43e-3
**GO**: **0010652**	positive regulation of cell communication by chemical coupling	143	6.43e-3
GO: 1903126	negative regulation of centriole-centriole cohesion	145	6.43e-3
GO: 0001927	exocyst assembly	146	6.43e-3
GO: 0090233	negative regulation of spindle checkpoint	148	6.43e-3
GO: 0060138	fetal process involved in parturition	149	6.43e-3
GO: 0048211	Golgi vesicle docking	150	6.43e-3
GO: 0070676	intralumenal vesicle formation	151	6.43e-3
GO: 0038016	insulin receptor internalization	152	6.43e-3
**GO**: **0014740**	negative regulation of muscle hyperplasia	193	1.28e-2

The annotations are the same as in Table 1.

Term combination p = 9.787e-22.

**Table 4:** The enrichment analysis result of CEA on renal cell carcinoma dataset.

GO ID	Description	Rank	p-values
**GO**: **0090260**	negative regulation of retinal ganglion cell axon guidance	39	3.34e-5
GO: 2000054	negative regulation of Wnt signaling pathway involved in dorsal/ventral axis specification	41	3.34e-5
GO: 0072027	connecting tubule development	236	5.82e-3
**GO**: **1900011**	negative regulation of corticotropin-releasing hormone receptor activity	240	5.82e-3
GO: 0097273	creatinine homeostasis	249	5.82e-3
GO: 0032972	regulation of muscle filament sliding speed	253	5.82e-3
GO: 0043438	acetoacetic acid metabolic process	254	5.82e-3
**GO**: **0002575**	basophil chemotaxis	256	5.82e-3
GO: 2000287	positive regulation of myotome development	260	5.82e-3
**GO**: **1901003**	negative regulation of fermentation	264	5.82e-3

The annotations are the same as in Table 1.

Term combination p = 8.366e-28.

For the lung cancer dataset, CEA uniquely identified two terms (GO: 0015014 and GO: 2000832). GO: 0015014 (heparan sulfate proteoglycan biosynthetic process, polysaccharide chain biosynthetic process) can be identified under another parameter combination of GenGO. Another term GO: 2000832 is related with the regulation of steroid hormone secretion. Researchers have shown that the female sex is a favorable factor in lung cancer prognostics, which indicates that the steroid hormone is closely related with lung cancer^22^.

For the ulcerative colitis dataset, CEA identified two GO terms (GO: 2000533 and GO: 0055059) that other methods failed to find. With regard to GO: 0055059 (asymmetric neuroblast division), asymmetric neuroblast division was observed with overexpression of Bazooka/Par-3 (Baz), a key regulator of cell polarity in neuroblasts. Protein phosphatase 2A (PP2A) dephosphorylates Baz at the conserved serine residue. Loss of PP2A function leads to complete reversal of polarity in neuroblasts^23,24^. The somatic mutations of the PP2A Aα and Aβ subunits have been reported in colon cancer^25,26^.

For the cervical carcinogenesis dataset, two terms GO: 0010652 (positive regulation of cell communication by chemical coupling) and GO: 0014740 (negative regulation of muscle hyperplasia) were uniquely identified by CEA. The former term involves the regulation of cell-cell communication. Studies have found that the mechanism of gap-junctional communication in cancer cells is impaired^27,28^. As for the latter GO term, muscle hyperplasia has been reported in cervical carcinogenesis patients^29^.

For the renal cell carcinoma dataset, most of the identified GO terms are not ranked at the top positions by the Fisher’s exact test, whereas the term combination is significantly enriched (p = 8.37e-28). Four GO terms (GO: 0090260, GO: 1900011, GO: 0002575, GO: 1901003) were uniquely identified by CEA. As for GO: 0002575 (basophil chemotaxis), the chemotaxis of basophil leukocyte, a type of immune cell, is directly correlated with cancer^30^. Studies support the hypothesis that the chemotaxis in stromal cells is an important component during cancer progression and metastasis^31^. GO: 1901003 (negative regulation of fermentation) is about the regulation of fermentation. Studies have found that the down-regulation of hypoxia inducible factor (HIF) is related to the Pasteur effect (an inhibiting effect of oxygen on the fermentation process)^32^ and the abnormal regulation of HIF is closely related with the tumorigenesis in renal cells^33–37^.

### CEA effectively reduces the redundancy of the identified terms

**Figure 2:**
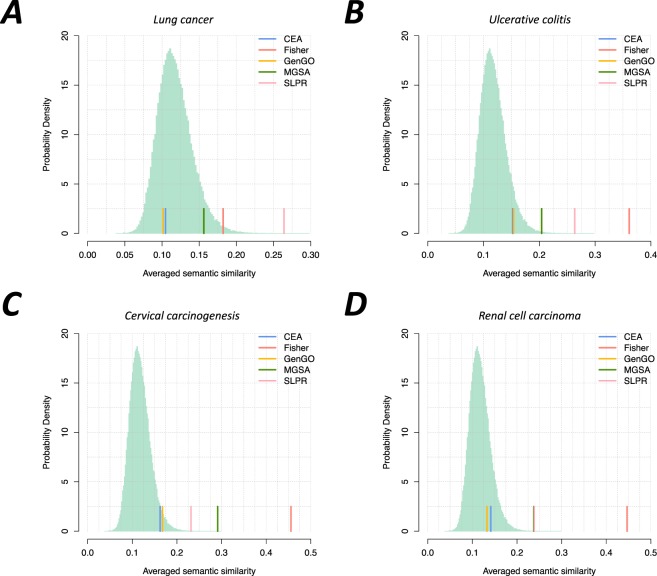
Comparison of the averaged semantic similarity scores. The light green histogram represents the background distribution of the averaged semantic similarity score. The semantic similarity score was computed using the R package GOSemSim^50^.

Highly similar and redundant functional enrichment analysis results often complicated the downstream efforts of researchers to interpret the underlying biological mechanisms present. We used the averaged semantic similarity score to measure the redundancy of the identified terms within each dataset, shown in Fig. 2 (see Materials and Methods for more details).

When compared with MGSA, SLPR, and the single-term-based approach, the averaged semantic similarity scores of CEA and GenGO are significantly reduced, indicating a lesser redundancy in the enrichment results. The scores of two combination-based approaches, CEA and GenGO, are very close to the mean value of background distribution, which implies the semantic similarities of terms in the identified term combination are very close to the random level. In contrast, the abilities of MGSA and SLPR to reduce the redundancy of identified terms are not obvious when compared to the above two methods. The Fisher’s exact test evaluates each term separately and does not consider the combination effects of identified terms to reduce the redundancy, which thereby generates a relatively high averaged semantic similarity.

**Figure 3:**
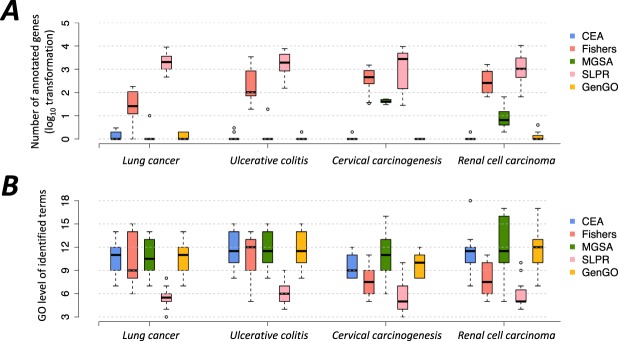
The boxplots of (**A**) the annotated gene number and (**B**) GO hierarchical levels of enriched terms identified by each method. The levels of identified GO terms were computed by using the R package topGO^51^.

The number of annotated genes and the GO hierarchical levels of enriched terms identified by each method are shown in Fig. 3. Terms with higher levels in the GO hierarchical structure (the root term is defined as level 0) are commonly specific terms that annotate a lower number of genes. Therefore, these two metrics can partly reflect the sources of the redundancy or similarity of identified terms.

In the box plots, CEA and GenGO share a similar tendency in all four datasets. The enriched terms identified by these two methods have relatively lower numbers of annotated genes and higher GO levels, which is consistent with the lower semantic similarity scores as shown in Fig. 2. The Fisher’s exact test and SLPR, on the contrary, are prone to identify several general terms with larger sizes and lower levels in the GO structure. We also found an interesting result for MGSA. The terms identified by MGSA are often in higher levels of GO structure, but they can also annotate larger numbers of genes. This phenomenon supports our initial design of the evaluation framework for gene set enrichment analysis methods, namely that we should evaluate one specific method from a comprehensive perspective rather than using some partial criteria.

Generally speaking, given an active gene list, each enriched term that is individually identified by the single-term-based approach such as the Fisher’s exact test contains some valuable information of the underlying dataset. When the redundancy of identified terms is reduced, it is expected that the important information contained in every enriched term should be kept as much as possible. For this purpose, we tested whether the term combination identified by CEA could serve as a representation for the enriched terms identified by the single-term-based method.

**Figure 4:**
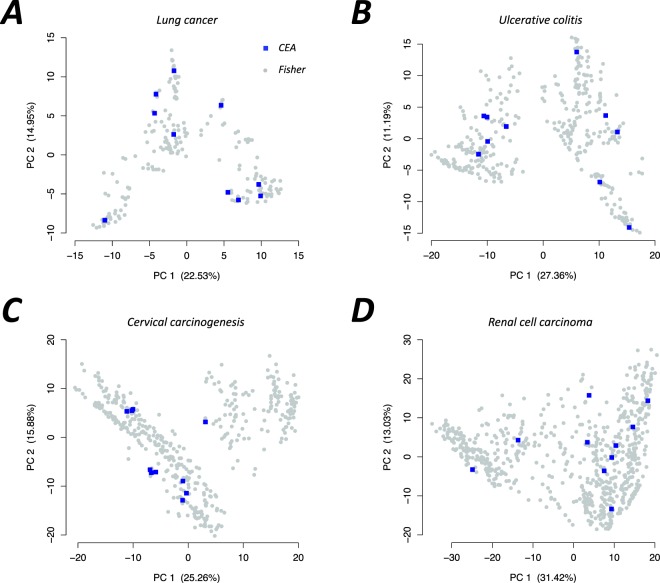
The representative relationship of the CEA terms and the single enriched terms. The PCA was performed basing on the semantic similarity matrix. The numbers in parentheses show the percentages of the contributions of the first and the second principal components, respectively.

Based on the semantic similarity score between each pair of enriched terms identified by CEA and the Fisher’s exact test, principal component analysis (PCA) was applied to visualize their relationship, as shown in Fig. 4. The PCA results show that the CEA terms (blue square points) are uniformly distributed among the single enriched terms (gray circular points) in all four datasets. In fact, we did not restrict the searching of term combination on the single enriched terms in the CEA algorithm. The CEA method naturally reveals the representative terms for the single enriched terms. In other words, CEA greatly reduces the redundancy but retains the important information to a high degree. This is helpful in the exploration of the underlying pathogenesis of complex diseases.

### CEA provides a landscape for systematically evaluating combination-based functional enrichment analysis methods

**Figure 5:**
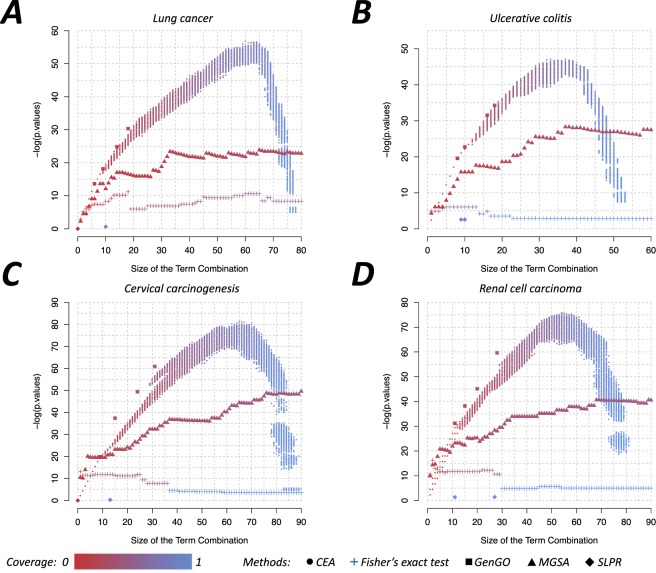
The landscape of combination-based functional enrichment analysis. The node color is correlated to the coverage of identified term combinations.

In this paper, we unified the combination-based functional enrichment analysis into a multi-objective combinatorial optimization framework. Benefiting from this framework, the existing combination-based function enrichment methods can be evaluated and compared in a comprehensive perspective. We can intuitively visualize the landscape provided by CEA framework, as shown in Fig. 5.

For each identified term combination, we calculated the size of the term combination (i.e. number of terms in the combination), the enrichment p-value, and the coverage to measure the performance of each method. From the results we can see that CEA produces many solutions with different sizes, p-values, coverages and provides the whole landscape of term combinations; therefore, it is a good benchmark for evaluating combination-based functional enrichment analysis methods.

An obvious trend that can be observed in Fig. 5 is that the term combinations with more terms are prone to a higher coverage, and vice versa. But the enrichment p-value has a different tendency. The enrichment p-value (with negative logarithm transformation) raises first and then drops sharply, like a parabolic curve. The reason is that the terms added in the late stage are not individually enriched in order to further increase the coverage. That is, the coverage is improved at the sacrifice of the enrichment performance. This indicates to us that it is unrealistic to explain all genes in the active gene list by a few common functional terms. There are always some genes distinct from others, for example, genes introduced by experimental noises or other undesired factors.

As shown in Fig. 5, the enrichment performance of the Fisher’s exact test is worse than CEA and GenGO. This implies that it is not practical to construct the term combination by simply selecting the top ranked enriched terms from the results of single-term-based method. The top ranked terms of single-term-based methods might be redundant and similar to each other, therefore their contribution to the term combination are minimal, if not negative. As for MGSA, the combinations of their top ranked terms consistently possess a lower coverage, even if the term number is large. On the contrary, SLPR identifies enriched terms with high coverage but the overall enrichment p-value is not very significant, which explains how SLPR is prone to finding some general terms that are in the lower levels of the GO structure (Figs 2 and 3).

The combination-based method GenGO exhibits exceptionally good performance under all four parameter settings. For each dataset, GenGO successfully identified the term combinations of the most significant enrichment p-values in the solutions with the same size. However, it is difficult to control and predict the size of term combinations identified by GenGO. Conversely, the distinguishing characteristic of CEA is that it can produce many near-optimal solutions for every desired size, which might provide more insight to the underlying functional mechanisms and improve the efficiency of the downstream analyses.

## Discussion

In this paper, we proposed a novel statistical framework for assessing the performance of combination-based gene set functional enrichment analysis. Using this framework, we formulated the enrichment analysis as a multi-objective combinatorial optimization problem and developed the CEA method. CEA is an efficient computational tool for combination-based functional enrichment analysis and provides an effective benchmark to evaluate and compare the existing combination-based methods.

As a combination-based approach, CEA can significantly reduce the redundancy of enriched terms while successfully identifying the crucial functional terms and retaining useful, diverse and representative information of enriched terms. More importantly, since CEA outputs many near-optimal solutions of different sizes instead of single optimal solution of one size, it can clearly show the landscape of candidate term combinations, which can be further utilized to analyze the relationship between enriched functions. This landscape also provides an innovative framework and benchmark to assess and compare the existing combination-based methods in a comprehensive perspective. It is easy and flexible for users to filter the enriched term combinations using their specific requirements, including the size of combination, the p-value cutoff, and the coverage.

From the viewpoint of combinatorial optimization, the framework of CEA is closely related to the proposed objective function, constraint conditions, as well as the selected approximation algorithm. All of these parts can affect the performance of CEA. For example, a randomized greedy algorithm is used in the current CEA method to solve the multi-objective combinatorial optimization problem. The randomized greedy algorithm has low computational complexity, which ensures the rapid running time of CEA. Although the randomized greedy algorithm can obtain exact optimal solutions in many cases as shown in our previous study^38^, its optimality is not guaranteed. In the datasets of cervical carcinogenesis and renal cell carcinoma, as shown in Fig. 5C,D, the solutions identified by GenGO have lower p-values than any solutions of CEA with the same size, which indicates that the solutions found by CEA are not exact Pareto-optimal. The performance of CEA could be further improved by increasing the parameters *d* and *T*, at the cost of much more computation time. Designing a better algorithm with lower approximation ratio and computational complexity is a challenge for future research.

Currently, there is no size constraint in the CEA model, which results in the CEA method being prone to identify more specific terms to cover the active genes. It is also the reason why the p-values of solutions with small sizes (e.g. less than 5) are even worse than that of the Fisher’s exact test. We may overcome this bottleneck by introducing a size constraint to the original optimization model. Using the information of biological networks have been successfully applied in many fields of bioinformatics^39–42^. Compared with traditional enrichment analysis, many computational approaches that integrate biological networks, such as network ontology analysis (NOA)^43^, network enrichment analysis (NEA)^44^, EnrichNet^45^ and NetGen^46^, have been developed to significantly improve the performance of enrichment analysis. Appropriately exploiting the resource of biological networks may further improve the performance of CEA. For example, NetGen used an idea that the influences of the active genes can propagate by their neighbors in the network. Inspired by NetGen, we can first extend the annotated gene set for each term by a similar network propagation step, and then perform the approximation algorithm in CEA.

The current framework of CEA is established based on applying the Fisher’s exact test to evaluate the overall enrichment of each term combination. Actually, as a competitive gene set test^47^, the Fisher’s exact test is prone to share the sensitivity to inter-gene correlation, which is nonnegligible in common enrichment tests^48^, and thereby increases the false positive rate of identified terms. The performance of CEA may be further improved by taking inter-gene correlation into consideration. How to properly integrate the information of inter-gene correlation will be another goal in our future research.

In this paper, we did not conduct a simulation study to compare the performances of combination-based methods. In a simulation study, the truly active gene list is known, and the gene expression data is generated according to some generative model, therefore the performance of each method can be assessed by objective quantitative criteria. But the performance of a particular method in a simulation study is closely related to whether its hypothesis fits the generative model. Since each method has its subtle mathematical model and different underlying hypotheses regarding the active gene list, these kinds of comparisons will never be fair to all methods. For example, SLPR is the best if the gene-level statistics have an additive association with gene set activity, but if the association is non-additive SLPR’s performance is not comparable to other methods based on non-additive assumption^19^. Most important, the complexity of real biological data makes it difficult to construct the best hypothesis to build an unbiased simulation of real world. Therefore, instead of evaluating combination-based enrichment analysis methods in a simulation study based on particular hypotheses, we tried to explore and compare the enrichment analysis results in multiple dimensions to reveal the characteristics of different methods. We believe the comprehensive comparison of results on real datasets can provide much more insights into the problem as well as guidance for real applications.

In real applications, CEA is not restricted to homo sapiens and GO annotation but can also be applied on any other species and functional annotations, such as KEGG pathway, OMIM or MSigDB. CEA has been implemented in the R package CopTea, which can be readily installed and used in R. CEA performs the gene set functional enrichment analysis from a different perspective and is a complementary tool to existing methods. We believe that CEA will have a widespread application in bioinformatics.

## Materials and Methods

### Statistical significance of term combinations

Most single-term-based methods utilize a statistical test such as the Fisher’s exact test to evaluate the enrichment of a functional term. Mathematically, each functional term can be represented as a set of genes annotated by this term. The statistical test calculates a p-value, i.e., the probability of observing the same or larger overlap between the set of genes annotated by the term and the active gene list produced by chance. Smaller p-values indicate more significant enrichment. All candidate terms are sorted according to their p-values, and the terms with multiple testing adjusted p-value smaller than a threshold (e.g. FDR = 0.05) are filtered as the output.

To establish a similar evaluation framework for combination-based approaches, we first construct a pseudo composite term for each term combination. This pseudo term consists of all genes that is annotated by at least one term in the term combination. Mathematically, the pseudo composite term is represented by the union of all terms (i.e. gene sets) in the corresponding term combination. In this way, the statistical significance of a term combination can be assessed by applying a statistical test such as the Fisher’s exact test on the pseudo composite term.

In the single-term-based approaches, it is computationally efficient to calculate the p-values for all terms. However, the computation of p-values for all term combinations becomes intractable in combination-based approaches. The number of possible term combinations exponentially increases with the number of terms. This phenomenon is called combinatorial explosion in mathematics. Therefore, it is impossible to enumerate and assess all possible term combinations. Even we restrict the combination size (i.e., the number of terms included in a term combination), the computation time is still unacceptable for real applications, in which there are often more than tens of thousands functional terms. To address this issue, we formulated the problem of identifying the most enriched term combination into an equivalent multi-objective optimization problem, which is further solved by a combinatorial optimization algorithm.

### Multi-objective optimization model

**Figure 6:**
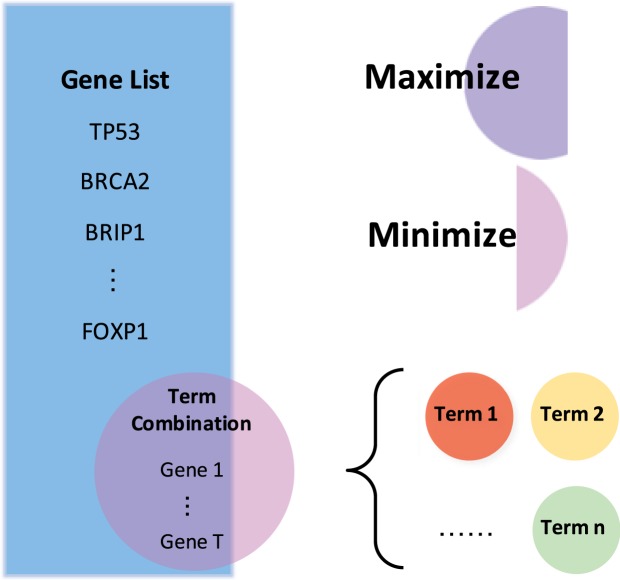
The sketch map of the multi-objective combinatorial optimization problem in combination-based functional enrichment analysis.

In the statistical framework proposed in previous section, the heavy computational burden can be dramatically reduced if we can only assess a small number of possible term combinations instead of all possible term combinations. In order to figure out a small scope for searching the most enriched term combination, we proposed a multi-objective combinatorial optimization problem as illustrated in Fig. 6.

The multi-objective combinatorial optimization problem aims to identify a term combination that (i) maximizes the overlapped genes between the active gene set *U*_*G*_ and the term combination (purple part); and (ii) minimizes the non-overlapped genes annotated by the term combination (pink part). The multi-objective combinatorial optimization problem can be formulated as follows:

$$\begin{array}{cc}{\rm{\max }} & t(X)\\ {\rm{\min }} & S(X)-t(X)\end{array}$$

where *X* is the term combination, *S*(*X*) is the number of annotated genes in the term combination *X*, *t*(*X*) is the number of overlapped genes between *U*_*G*_ and the term combination *X*.

Obviously, the two objectives of the multi-objective optimization problem are conflicting and cannot be simultaneously optimized. A solution of multi-objective optimization problem is called Pareto optimal if none of the objectives can be further improved without making other objectives worse off. It can be theoretically proved that the term combination with the smallest enrichment p-value is a Pareto-optimal solution. Therefore, we can only perform the statistical test in the Pareto-optimal solutions of the above multi-objective optimization problem. Generally, there might exist a number of Pareto optimal solutions, possibly infinite. Since it is a combinatorial optimization problem and 0 ≤ *t*(*X*) ≤ |*U*_*G*_|, the number of Pareto optimal solutions is the same as the size of active gene set, which is often small.

In order to solve the above multi-objective optimization problem, we further transformed it into a series of combinatorial optimization problems as follows:

$$\begin{array}{c}\mathop{{\rm{\min }}}\limits_{X}S(X)-t(X)\\ {\rm{s}}.{\rm{t}}.\,t(X)\ge \alpha |{U}_{G}|\end{array}$$

By varying the parameter *α* ∈ [0, 1], introduced to control the degree of coverage, we can obtain all Pareto-optimal solutions of the original multi-objective optimization problem. Unfortunately, even for a fixed *α*, the exact optimal solution of this combinatorial optimization model is still difficult to solve. The special case when *α* = 1 is a variant of the famous set cover problem, named enrichment set cover problem (ESCP), and has been proven to be NP-hard^38^. Four approximation algorithms were designed and theoretically analyzed in our previous work^38^. All of them could successfully find the optimal solutions in almost all simulated examples of small to moderate size based on the real datasets.

### Combination-based enrichment analysis (CEA) method

Taking both the practical performance and the computation complexity into consideration, we developed the Combination-based Enrichment Analysis (CEA) method basing on the IMPROVED GREEDY algorithm^38^ to execute the enrichment analysis.

Given an active gene set *U*_*G*_, denote *U*_*O*_ the set of genes not in *U*_*G*_, and *U* is the set of all genes in one species, i.e. *U* = *U*_*G*_ ∪ *U*_*O*_. ${\mathscr{S}}=\{{S}_{1},{S}_{2},\cdots ,{S}_{m}\}$ is the set of all candidate functional terms. $M=\{1,2,\cdots ,m\}$ is the index set of ${\mathscr{S}}$. $X\subseteq M$ is the index set of the selected terms. The coverage of terms in *X* is defined as:

$$C(X)=\frac{|(\mathop{\cup }\limits_{i\in X}\,{S}_{i}){\cap }^{}{U}_{G}|}{|{U}_{G}|},$$

that is the proportion of the covered active genes. Denote the weight of element *e* in *U*_*O*_ as:

$$w(e)=\frac{1}{|{N}_{e}|},$$

where ${N}_{e}=\{i|e\in {S}_{i}\}$ is the coverage frequency of element *e*. Thus, the weight of set *S* in ${\mathscr{S}}$ is defined as:

$$w(S)=\sum _{e\in S\cap {U}_{O}}w(e),$$

which is equal to the sum of the weights of the elements cover by *S*, that not in the active gene set *U*_*G*_.

The pseudo-code of CEA is as follows:

**Algorithm:**
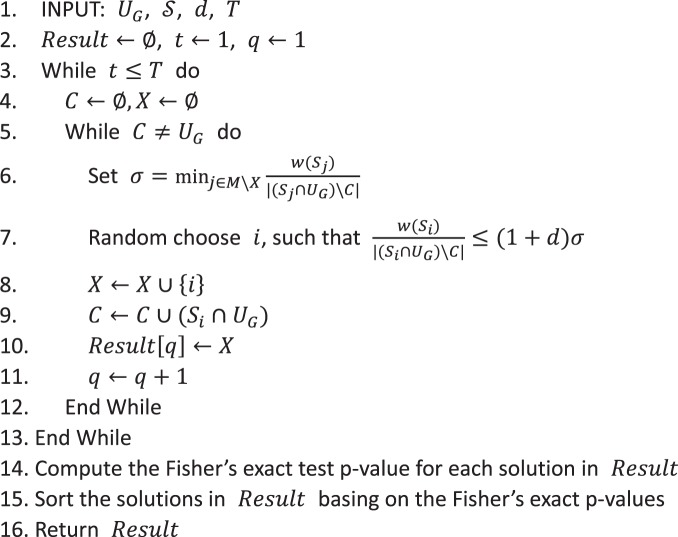
Combination-based Enrichment Analysis (CEA).

The inputs of CEA are the active gene set *U*_*G*_, the candidate term set ${\mathscr{S}}$ and two model parameters, the randomization parameter *d* and the repeat times *T*. The output of CEA is a set of near-optimal solutions obtained in each iteration which have different coverages and p-values. The users can filter the solutions based on the pre-set criteria, e.g. the threshold of p-value or the desired size of term combinations.

The whole framework of CEA is based on a greedy algorithm. We used the weighted penalty for annotating extra active genes, i.e., $\frac{w({S}_{i})}{|({S}_{i}\cap {U}_{G})\,\backslash \,C|}$ (in step 6 and 7), as the cost-effectiveness of term *i*. The algorithm would prefer the term which can annotate more extra active genes whereas annotate less genes in *U*_*O*_. Notably, the novel part of CEA method is that we added a randomization parameter *d* into the greedy algorithm. In each iteration, we randomly select a term with cost-effectiveness no more than (1 + *d*) times of the minimum (*σ*), instead of directly selecting the term with minimum cost-effectiveness. When *d* = 0, the algorithm degenerates to the deterministic greedy algorithm. The randomness of CEA can help the algorithm to escape from the local minimums. By repeatedly running the randomized greedy algorithm, the chance that CEA finds better solutions is significantly higher than the deterministic greedy algorithm. Larger *d* can increase the variance of solutions, therefore improving the quality of the best solutions. However, the larger the value of *d*, the more repeat times are needed. The performance analysis of CEA with different *d* and *T* can be found in Fig. S1 in Supplementary Materials. Based on our analysis, the default values of these two parameters are selected as *d* = 1 and *T* = 500.

### Alternative methods description

In order to evaluate the proposed enrichment analysis method CEA, three state-of-the-art methods (GenGO, MGSA and SLPR) and a popular single-term-based approach (Fisher’s exact test) were employed for comparison purpose.

GenGO is a generative probabilistic method proposed by Lu *et al*.^15^, which identifies the enriched GO terms by maximizing the log-likelihood conditioned on the set of active genes. This method discourages the identification of highly overlapping GO terms and is effective both on microarray and ChIP-chip data. There are three parameters, *p*, *q* and *α* in GenGO. In order to evaluate its performance more comprehensively, we used the following four parameter combinations for GenGO to obtain four different final results.

$$\begin{array}{c}p=0.8,\,q=0.001,\,\alpha =3\\ p=0.5,\,q=0.001,\,\alpha =3\\ p=0.8,\,q=0.01,\,\alpha =3\\ p=0.5,\,q=0.01,\,\alpha =3\end{array}$$

MGSA^14^ uses the probabilistic inference to identify the active terms, which are embedded in a three-layer Bayesian network. We used the source code with the default parameters in ‘mgsa’ package which is compiled in Bioconductor^49^. We set the number of different runs of the MCMC sampler (parameter *restarts*) to 10 and each run of MCMC has 1e7 steps (parameter *steps*) to get the final result, which is a term list sorted by the estimated posterior probability.

SLPR^19^ identifies the enriched term set via LASSO penalized regression. In our real data application, we followed the procedures as described in ‘Real data analysis design’ in SLPR to obtain the gene-level test statistic only for active genes. In detail, the statistic of active gene is defined as the smaller absolute value of the estimated 95% confidence interval in two-sided Student’s t-test of disease and normal samples. There are two different ways to execute SLPR. We kept both the term sets identified via specified output numbers (parameter *lambda*.*via*.*CV* = *FALSE* and *num*.*predictors* = 10) and via seeking the optimal lambda in cross validation.The Fisher’s exact test^21^ is widely used in many single-term-based approaches for identifying the enriched functional terms. In this paper, we used the Fisher’s exact test to evaluate each term separately and thereby got a sorted term list by sorting the test p-value of each term in ascending order.

Due to the complexity of real datasets, we actually do not know which the best model parameters for each method are. To make the results comparable, we analyzed the term sets identified by each method whose size is close to 10. For CEA, we used the most enriched term combinations with 10 terms. As for MGSA and the Fisher’s exact test, we used the most enriched top 10 terms. In the final comprehensive evaluation and comparison of different methods, we reported and visualized all the candidate term sets identified by each method (no size restriction). The Fisher’s exact test and MGSA both output sorted term lists, we separately evaluated the top enriched term sets with different sizes.

### Semantic similarity-based analysis

The GO semantic similarity has been widely used in bioinformatics applications. It provides a criterion to measure the redundancy between GO terms. Generally speaking, a lower semantic similarity score indicates a lower redundancy between two GO terms. In this study, we applied the averaged GO semantic similarity score to measure the redundancy of GO terms in the identified term combination. The semantic similarity score was computed using the R package GOSemSim^50^ compiled in Bioconductor^49^. Mathematically, the averaged GO semantic similarity score for a term set *S* is defined as:

$$ASS({\mathscr{S}})={(\begin{array}{c}n\\ 2\end{array})}^{-1}\sum _{1\le i < j\le n}\,score({S}_{i},\,{S}_{j})$$

where ${\mathscr{S}}=\{{S}_{1},{S}_{2},\cdots ,{S}_{n}\}$ is a set of interested GO terms and *n* is the size of ${\mathscr{S}}$. *score* (*S*_*i*_, *S*_*j*_) is the semantic similarity score between terms *S*_*i*_ and *S*_*j*_.

Using the above formula, we first computed the semantic similarity scores of the identified term sets derived by different enrichment approaches. Additionally, we obtained a background distribution of the averaged semantic similarity scores to make the results more comparable. In each iteration, we randomly sampled a term set ${\mathscr{S}}^{\prime}$ from all candidate GO terms (not restricted only to identified term sets). ${\mathscr{S}}^{\prime}$ contains the same number of terms as the identified term sets, and the semantic similarity score $ASS({\mathscr{S}}^{\prime} )$ is computed. The above procedure is repeated for 100,000 times to get the background distribution of the semantic similarity score.

The GO hierarchical levels can reflect the specificity of identified terms. The level of a term is defined as the length of longest path to the root term in the GO hierarchical structure. Terms with higher levels in the GO hierarchical structure are more specific terms that annotate a lower number of genes. The levels of identified GO terms were computed by using the R package topGO^51^ in Bioconductor^49^.

### Datasets

The proposed enrichment analysis method CEA and the alternative methods were evaluated and compared on the GO annotations. The GO annotation dataset was derived from the R package *org*.*Hs*.*eg*.*db* provided in Bioconductor project. Only the enrichment analysis results on the biological process (BP) domain are summarized and shown in this paper.

The active gene lists used in the experiments were derived from four real human disease microarray datasets. These datasets were downloaded from the Gene Expression Omnibus (GEO^52,53^) repository with accession number GSE4115, GSE11223, GSE9750, GSE36895, respectively. The selection criteria and the details of data preprocessing can be found in Supplementary Materials.

Each active gene list *U*_*G*_ was generated from the corresponding microarray dataset by the following steps:

1. For each gene, a p-value of the Student’s t-test executed on the disease and the control groups of samples was obtained.
2. The candidate genes were sorted in ascending order of the Student’s t-test p-values.
3. The top 100 genes were defined as the differentially expressed genes.
4. The differentially expressed genes with at least one GO annotation were used as the active genes of each dataset.

The details of four active gene lists were listed in Supplementary Materials.

## Electronic supplementary material

Supplementary Materials

## Data Availability

CEA has been implemented in the R package CopTea, which is publicly available at GitHub (http://github.com/wulingyun/CopTea/), and readily installed and used in R. The codes used in this paper and the additional materials are available at http://doc.aporc.org/wiki/CEA.
