# Supplementary material for: CEA: Combination-based gene set functional enrichment analysis
Source: Sci Rep. 2018 Aug 30;8:13085. doi: 10.1038/s41598-018-31396-4 (PMC6117355; doi:10.1038/s41598-018-31396-4)
Supplement: Supplementary file 1 — Supplementary Materials [file 41598_2018_31396_MOESM1_ESM.pdf]

Supplementary Materials

CEA: Combination-based gene set functional enrichment analysis

Duanchen Sun[1,2], Yinliang Liu[1,2], Xiang-Sun Zhang[1], Ling-Yun Wu[1,2,*]

[1]IAM, MADIS, NCMIS, Academy of Mathematics and Systems Science, Chinese Academy of Sciences, Beijing 100190, China

[2]School of Mathematical Sciences, University of Chinese Academy of Sciences, Beijing 100049, China

1. The performance of CEA using different d and T

In the CEA method, we introduced a randomization parameter d, which could help the algorithm to escape the local minimum. Larger d will increase the variance of solutions identified by the algorithm. That is, the probabilities to find better solutions as well as worse solutions are both increased. Therefore, the algorithm need repeat sufficient times in order to find better solutions. Larger d often requires more repeat times of algorithm. In this section, we conducted a simulated experiment to explore the effects of parameters d and T to the final result of CEA.

The simulated datasets were extracted from the biological process (BP) domain of GO. We simulated the active gene lists using a more appropriate approach (see below). Our simulation was based on the biological assumption that the active gene list derived from a specific biological experiment usually has close relationship with several biological processes.

The original annotation matrix of BP domain, derived from the Bioconductor R package *org.Hs.eg.db*, contains 14614 genes and 13226 terms. We first filtered the terms and kept the terms that annotate 50 to 100 genes as candidate terms. Namely, too general or specific terms were filtered out. This preprocessing could avoid the final results have a large variance and reduce the total computation time.

In the experiment, the following values of parameters d and T were considered:

$$d = \{0, 0.01, 0.1, 1, 10\},$$

$$T = \{1, 10, 50, 100, 200, 500\},$$

The detailed procedure of our exploration is as follows:

1) Randomly select one term from the candidate terms into the current term combination, until the number of annotated genes is no less than 200.
2) Randomly select 100 annotated genes from the genes annotated by the current term combination as the active gene list.
3) For each d and T, execute the CEA algorithm to compute the enriched term combinations using the given active gene list.
4) Sort the identified term combinations based on the p-values of the Fisher's exact test.
5) Record the mean value of $-log_{10}(p)$ of the top 30 enriched term combinations.
6) Repeat the above procedure for 100 times to achieve a robust result.

The final results were shown in Figure S1.

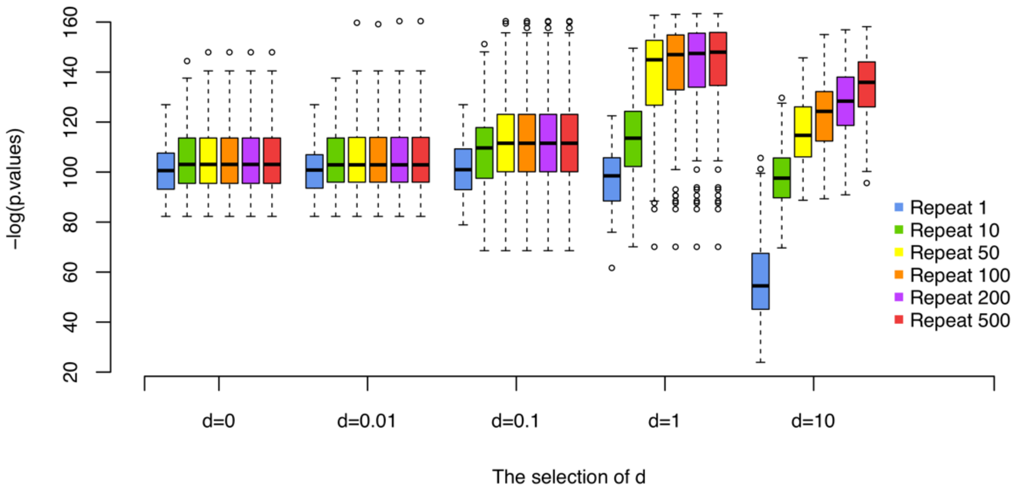

Figure S1. The performance of CEA using different $\boldsymbol{d}$ and $\boldsymbol{T}$. For each d, a group of boxplots for each repeat times T was plotted. The performance was evaluated by the negative logarithmic transformation of p-values.

As expected, the results clearly showed that the performance of CEA can be significantly improved by introducing the randomization parameter d and CEA needs more repeat times to achieve a desired performance when d increasing.

Generally, for a fixed d, the performance of CEA will be improved if more repeat times is executed. The performance of CEA would be very poor when the repeat times is insufficient (e.g. $d = 10$ and $T \leq 10$). However, the performance cannot be improved infinitely by increasing the repeat times T. For enough large T, the marginal improvement becomes very small. Therefore, the users should balance the trade-off between the performance and the computation time.

We can roughly estimate an appropriate repeat times $\tilde{T}$ for a given d from the above results. It seems that $\tilde{T} = 500d$ would be enough for good performance. In this paper, we selected $d = 1$ and $T = 500$ as the default values to execute the CEA algorithm.

2. The preprocessing of gene expression datasets

In this work, we used real gene expression datasets of human complex diseases to test whether the term combinations identified by CEA are meaningful and closely related to the corresponding disease. The selection of gene expression datasets is based on the following criteria:

1) *Homo sapiens* organism disease;
2) Published (submission date) in recent ten years;
3) A balanced number of case and control samples and the total number is at least 50;

According to these criteria, four gene expression microarray datasets of human complex diseases were selected from the Gene Expression Omnibus repository [1] (http://www.ncbi.nlm.nih.gov/geo/), with accession number GSE4115, GSE11223, GSE9750, GSE36895, respectively, for real datasets analyses.

As for lung cancer dataset (GSE4115 [2]), we combined the original primary and prospective datasets, which made a total of 97 and 90 smokers with and without lung cancer, respectively. For ulcerative colitis dataset (GSE11223 [3]), we only used the uninflamed samples in each cohort, which made a total of 66 ulcerative colitis patients and 69 healthy control donors. All samples of the cervical carcinogenesis dataset (GSE9750 [4]) were kept. As for renal cell carcinoma (GSE36895 [5]), the paired expression profiles of 23 clear-cell RCC patients and their related normal cortex were used for further analysis.

For all expression datasets, we averaged the expression values of the probe sets mapping on the same gene. The summaries of the preprocessed datasets are shown in Table S1.

Table S1: The summary of gene expression datasets used in our work.

Dataset	accession number	#disease	#normal	#genes
lung cancer	GSE4115	97	90	12493
ulcerative colitis	GSE11223	66	69	10506
cervical carcinogenesis	GSE9750	33	24	12494
renal cell carcinoma	GSE36895	23	23	20108

3. Active gene lists used in the real data analysis

For each microarray dataset, we generated a representative active gene list after preprocessing the original dataset. The detailed procedure of generating the active gene list is introduced in the main text.

The active gene lists used as the input of each enrichment analysis method are listed as follows:

Lung cancer (81):

SLC5A1 PRUNE ATP8B1 NSUN3 HDGFRP3 STK38 AGPS TRIM36 DCLRE1C BTD RPL35A SOX9 DND1 C6 TSR1 NNT ZNF160 TFE3 HTRA1 ADH6 PDE8B ZNF611 U2AF2 ECD TMEM110 GOSR2 GTF2H3 SUGP2 MOCS2 PPP2R2D RPL18 P2RX4 NEDD9 SLC4A4 ADK PGF CRY1 EXT2 NOTCH2NL EIF2B3 CORO2A FGF14 DMD DLAT DIP2A USP46 HAUS2 ALPK1 MAN1A2 PPM1D CEP57 DAPP1 PRDX2 NPFFR1 STX3 LAT FBXO9 WWC3 TGDS ARID5A UBQLN4 GNPDA1 RHOQ TNFRSF1A CPE ODF2 PYGB FUT8 ZFR NUDT4 TXN DNAJC6 MTPAP RRAGB ABHD17B IL13RA1 MSH6 MYO1C UNC93B1 MFSD11 KDELR3

Ulcerative colitis (56):

PLCB3 ELL MAPKAPK2 DOCK7 DOHH STK25 TBXA2R INPPL1 C6orf120 APOC1 CEP290 STK35 LARP1 GTF2H5 PPP1R14B SBF1 DIRC2 BRD4 AXIN1 INSR SKIV2L PRCP B3GALT5 TAF12 VPS52 RPS29 ZNF304 C14orf2 ITGA3 GAS6 ARF6 SPSB1 USP54 SLC2A8 GCA CCL11 SERPINF1 FBXL12 TBC1D2B MAN2A1 HIST1H2BN GNB2 ACYP2 ARAF BLVRA HOMER3 PUS1 ACSM1 ADAL C3orf33 GBE1 COMP OXSR1 MVD MLXIP DDX6

Cervical carcinogenesis (94):

PITPNA ZDHHC3 GJA1 SYNGR1 KCTD15 ESR1 AHNAK TRPS1 CDKN2A KANK1 KRT13 KIF18B SYPL1 NAGK MCM6 LMBRD1 UBE2E1 CHMP2B SPRR3 USO1 GINS2 RPL10A NEK2 MCM2 ZNF586 DNMT1 POLD1 RAD54L GOLGA4 CRYL1 GINS1 RPS12 SKP1 SLC24A3 UBE2C MAP2K4 CHAF1B PLCD1 KNTC1 PRDM2 MCM5 ZNF415 TK1 KIF4A KIF2C AURKA CAPN7 TP53AIP1 CCNF LPAR6 SNX3 RPS6KA1 ATP6V1F LAPTM4A PPP2R5A ITM2B DUSP1 NUP62 ATP13A2 RPL29 ATP10D CENPF USP46 LIG1 ARHGAP10 STX7 BBOX1 KLF4 CLCA4 SPAG5 TMEM9B DSC2 RYR1 LANCL1 SYNGR3 AVPR1B TPX2 PSMC3IP SASH1 MAPK10 CDC20 CDT1 CDC45 GIGYF2 TRIM13 TIMELESS GALR3 SLC15A3 IL17RC CDC6 CLCN3 RALB DTL PERP

Renal cell carcinoma (85):

NPHS2 SPAG4 UMOD SFRP1 FGF1 SLC12A1 EGLN3 IGFBP3 ATP6V0D2 HK2 CALB1 GGT6 CWH43 CLDN8 HILPDA HEPACAM2 LPPR1 ATP6V0A4 ACSF2 ANGPTL4 SCNN1G PTH1R CLIC5 FAM3B CLCNKB ENO2 SLIT2 PPAPDC1A PRKCDBP FUT11 CRHBP TMPRSS2 PLCXD3 SAP30 SLC47A2 PTGDS HS6ST2 FXYD4 ATP6V1G3 TYRP1 TCEAL2 TNNC1 DMRT2 CNTN1 HPD SER INA5 KNG1 GPD1L STAP1 C5 CAV1 PDK1 PTPRO RASL11B SLC26A7 GAS1 CAV2 TFAP2B LDHA NPHS1 TCF21 DDB2 SLC2A12 PACRG KCNJ10 DIO1 DACH1 ARHGEF26 GPC3 BMPR1B SEC61G NRK ALDOA VEGFA MUC15 EIF4H CA10 MAN1C1 COL4A6 SOSTDC1 SOST ATP6V1C2 ATP6V1B1 ANGPTL1 FABP5

4. Detailed results of GenGO, MGSA and SLPR

In this section, we listed the enriched GO terms identified by GenGO, MGSA and SLPR. Similar to the tables shown in main text, we highlighted the corresponding uniquely terms identified by each method. The GenGO results can be found in Tables S2-S5.

Table S2. The enrichment analysis result of GenGO on lung cancer dataset. The Fisher's exact test p-values and ranks for each single GO term were also listed. The p-value of the term combination was shown at the bottom of the table. The boldfaces were the GO terms identified only by GenGO, compared with CEA, MGSA and SLPR.

GO ID	Description	Rank	p-values
GO: 0006175	dATP biosynthetic process	10	5.54e-3
GO: 0036071	N-glycan fucosylation	11	5.54e-3
GO: 0035772	interleukin-13-mediated signaling pathway	12	5.54e-3
GO: 0060149	negative regulation of posttranscriptional gene silencing	13	5.54e-3
GO: 0060517	epithelial cell proliferation involved in prostatic bud elongation	16	5.54e-3
GO: 0006043	glucosamine catabolic process	18	5.54e-3
**GO: 0009785**	blue light signaling pathway	28	1.11e-2
GO: 0072318	clathrin coat disassembly	34	1.11e-2
GO: 0021648	vestibulocochlear nerve morphogenesis	37	1.11e-2

*Term combination p=6.776e-19

Table S3. The enrichment analysis result of GenGO on ulcerative colitis dataset. The annotations are the same as in Table S2.

GO ID	Description	Rank	p-values
GO: 0010900	negative regulation of phosphatidylcholine catabolic process	47	3.83e-3
**GO: 0071307**	cellular response to vitamin K	48	3.83e-3
GO: 0038193	thromboxane A2 signaling pathway	52	3.83e-3
GO: 0021881	Wnt-activated signaling pathway involved in forebrain neuron fate commitment	53	3.83e-3
GO: 1900402	regulation of carbohydrate metabolic process by regulation of transcription from RNA polymerase II promoter	54	3.83e-3
GO: 1901407	regulation of phosphorylation of RNA polymerase II C-terminal domain	55	3.83e-3
GO: 0018874	benzoate metabolic process	56	3.83e-3
GO: 0006294	nucleotide-excision repair, preincision complex assembly	57	3.83e-3
GO: 0007439	ectodermal digestive tract development	92	7.65e-3
**GO: 0031119**	tRNA pseudouridine synthesis	99	7.65e-3

*Term combination p=1.914e-23

Table S4. The enrichment analysis result of GenGO on cervical carcinogenesis dataset. The annotations are the same as in Table S2.

GO ID	Description	Rank	p-values

GO: 0086042	cardiac muscle cell-cardiac muscle cell adhesion	139	6.43e-3
**GO: 0003294**	atrial ventricular junction remodeling	140	6.43e-3
GO: 1903126	negative regulation of centriole-centriole cohesion	145	6.43e-3
GO: 0001927	exocyst assembly	146	6.43e-3
GO: 0090233	negative regulation of spindle checkpoint	148	6.43e-3
GO: 0060138	fetal process involved in parturition	149	6.43e-3
GO: 0048211	Golgi vesicle docking	150	6.43e-3
GO: 0070676	intralumenal vesicle formation	151	6.43e-3
GO: 0038016	insulin receptor internalization	152	6.43e-3

*Term combination p=1.273e-20

Table S5. The enrichment analysis result of GenGO on renal cell carcinoma dataset. The annotations are the same as in Table S2.

GO ID	Description	Rank	p-values
GO: 0090259	regulation of retinal ganglion cell axon guidance	12	7.56e-7
GO: 2000054	negative regulation of Wnt signaling pathway involved in dorsal/ventral axis specification	41	3.34e-5
GO: 0070836	caveola assembly	75	1.99e-4
GO: 0072027	connecting tubule development	236	5.82e-3
**GO: 0051460**	negative regulation of corticotropin secretion	238	5.82e-3
GO: 0097273	creatinine homeostasis	249	5.82e-3
GO: 0032972	regulation of muscle filament sliding speed	253	5.82e-3
GO: 0043438	acetoacetic acid metabolic process	254	5.82e-3
GO: 2000287	positive regulation of myotome development	260	5.82e-3
**GO: 0006113**	fermentation	262	5.82e-3
**GO: 0060720**	spongiotrophoblast cell proliferation	265	5.82e-3

*Term combination p=6.439e-32

The terms identified by GenGO and CEA are largely overlapped. There are even several terms that uniquely identified by CEA and GenGO have closely relationships. For example, GO: 1900011 (negative regulation of corticotropin-releasing hormone receptor activity) and GO: 0051460 (negative regulation of corticotropin secretion) are co-occurring terms in renal cell carcinoma dataset. Specially, GO:1901003 (negative regulation of fermentation) is a child of GO:0006113 (fermentation)[6], which indicate that CEA could identify more specific terms.

The MGSA results can be found in Tables S6-S9.

Table S6. The enrichment analysis result of MGSA on lung cancer dataset. The Fisher's exact test p-values and ranks for each single GO term were also listed. The p-value of the term combination was shown at the bottom of the table. The boldfaces were the GO terms identified only by MGSA, compared with CEA, GenGO and SLPR.

GO ID	Description	Rank	p-values
**GO: 0006491**	N-glycan processing	2	1.33e-3
GO: 0006175	dATP biosynthetic process	10	5.54e-3

GO: 0036071	N-glycan fucosylation	11	5.54e-3
GO: 0035772	interleukin-13-mediated signaling pathway	12	5.54e-3
GO: 0060149	negative regulation of posttranscriptional gene silencing	13	5.54e-3
**GO: 0060965**	negative regulation of gene silencing by miRNA	14	5.54e-3
GO: 0060967	negative regulation of gene silencing by RNA	15	5.54e-3
GO: 0060517	epithelial cell proliferation involved in prostatic bud elongation	16	5.54e-3
GO: 0060784	regulation of cell proliferation involved in tissue homeostasis	17	5.54e-3
GO: 0006043	glucosamine catabolic process	18	5.54e-3

*Term combination p=7.656e-13

Table S7. The enrichment analysis result of MGSA on ulcerative colitis dataset. The annotations are the same as in Table S6.

GO ID	Description	Rank	p-values
**GO: 0034243**	regulation of transcription elongation from RNA polymerase II promoter	3	4.94e-5
GO: 0010900	negative regulation of phosphatidylcholine catabolic process	47	3.83e-3
**GO: 1900141**	regulation of oligodendrocyte apoptotic process	49	3.83e-3
**GO: 1900142**	negative regulation of oligodendrocyte apoptotic process	50	3.83e-3
GO: 0038193	thromboxane A2 signaling pathway	52	3.83e-3
GO: 0021881	Wnt-activated signaling pathway involved in forebrain neuron fate commitment	53	3.83e-3
GO: 1900402	regulation of carbohydrate metabolic process by regulation of transcription from RNA polymerase II promoter	54	3.83e-3
GO: 1901407	regulation of phosphorylation of RNA polymerase II C-terminal domain	55	3.83e-3
GO: 0018874	benzoate metabolic process	56	3.83e-3
GO: 0006294	nucleotide-excision repair, preincision complex assembly	57	3.83e-3

*Term combination p=1.768e-16

Table S8. The enrichment analysis result of MGSA on cervical carcinogenesis dataset. The annotations are the same as in Table S6.

GO ID	Description	Rank	p-values
**GO: 0006271**	DNA strand elongation involved in DNA replication	3	3.42e-11
**GO: 0022616**	DNA strand elongation	5	7.17e-11
**GO: 0030071**	regulation of mitotic metaphase/anaphase transition	18	7.62e-7
**GO: 1902099**	regulation of metaphase/anaphase transition of cell cycle	20	8.60e-7
**GO: 0031577**	spindle checkpoint	21	9.97e-7
**GO: 0007094**	mitotic spindle assembly checkpoint	27	4.79e-6
**GO: 0045841**	negative regulation of mitotic metaphase/anaphase transition	29	5.45e-6
**GO: 0071173**	spindle assembly checkpoint	30	5.45e-6
**GO: 1902100**	negative regulation of metaphase/anaphase transition of cell cycle	31	6.17e-6

**GO: 0051983**	regulation of chromosome segregation	47	3.87e-5

*Term combination p=3.011e-20

Table S9. The enrichment analysis result of MGSA on renal cell carcinoma dataset. The annotations are the same as in Table S6.

GO ID	Description	Rank	p-values
GO: 0007588	excretion	2	1.21e-10
GO: 0090259	regulation of retinal ganglion cell axon guidance	12	7.56e-7
**GO: 0031290**	retinal ganglion cell axon guidance	26	5.92e-6
**GO: 0072017**	distal tubule development	37	3.03e-5
GO: 2000054	negative regulation of Wnt signaling pathway involved in dorsal/ventral axis specification	41	3.34e-5
**GO: 1902667**	regulation of axon guidance	51	8.22e-5
**GO: 2000053**	regulation of Wnt signaling pathway involved in dorsal/ventral axis specification	55	9.99e-5
GO: 0070836	caveola assembly	75	1.99e-4
**GO: 0044332**	Wnt signaling pathway involved in dorsal/ventral axis specification	110	4.94e-4
**GO: 0001765**	membrane raft assembly	118	6.89e-4

*Term combination p=1.236e-22

The SLPR results can be found in Tables S10-S13.

Table S10. The enrichment analysis result of SLPR on lung cancer dataset. The Fisher's exact test p-values and ranks for each single GO term were also listed. The p-value of the term combination was shown at the bottom of the table. The boldfaces were the GO terms identified only by SLPR, compared with CEA, GenGO and MGSA.

GO ID	Description	Rank	p-values
**GO: 0071702**	organic substance transport	70	2.09e-2
**GO: 0009892**	negative regulation of metabolic process	179	4.43e-3
**GO: 0010629**	negative regulation of gene expression	430	1.05e-1
**GO: 0044087**	regulation of cellular component biogenesis	477	1.15e-1
**GO: 0050896**	response to stimulus	525	1.27e-1
**GO: 0044281**	small molecule metabolic process	552	1.36e-1
**GO: 0050789**	regulation of biological process	553	1.36e-1
**GO: 0006812**	cation transport	820	2.39e-1
**GO: 0048522**	positive regulation of cellular process	1823	8.19e-1
**GO: 0050790**	regulation of catalytic activity	1956	9.67e-1

*Term combination p=0.233

Table S11. The enrichment analysis result of SLPR on ulcerative colitis dataset. The annotations are the same as in Table S10.

GO ID	Description	Rank	p-values
**GO: 0006796**	phosphate-containing compound metabolic process	1	1.06e-5

**GO: 0018193**	peptidyl-amino acid modification	4	6.43e-5
**GO: 0019220**	regulation of phosphate metabolic process	11	5.07e-4
**GO: 0044710**	metabolic process	14	6.34e-4
**GO: 0031329**	regulation of cellular catabolic process	15	6.95e-4
**GO: 0044712**	catabolic process	18	8.25e-4
**GO: 0008217**	regulation of blood pressure	43	2.78e-3
**GO: 0019538**	protein metabolic process	87	7.32e-3
**GO: 0043170**	macromolecule metabolic process	158	1.34e-2
**GO: 0051239**	regulation of multicellular organismal process	360	4.37e-2

*Term combination p=2.833e-3

Table S12. The enrichment analysis result of SLPR on cervical carcinogenesis dataset. The annotations are the same as in Table S10.

GO ID	Description	Rank	p-values
**GO: 0044267**	cellular protein metabolic process	334	2.62e-2
**GO: 0032526**	response to retinoic acid	346	2.94e-2
**GO: 0009314**	response to radiation	498	5.66e-2
**GO: 0031424**	keratinization	908	1.65e-1
**GO: 0018149**	peptide cross-linking	941	1.76e-1
**GO: 0065007**	biological regulation	1014	2.02e-1
**GO: 0044710**	metabolic process	1095	2.28e-1
**GO: 0009913**	epidermal cell differentiation	1124	2.42e-1
**GO: 0050896**	response to stimulus	1193	2.71e-1
**GO: 0009611**	response to wounding	1396	3.69e-1
**GO: 0007275**	multicellular organism development	1448	3.99e-1
**GO: 0032502**	developmental process	1489	4.18e-1
**GO: 0048513**	animal organ development	1911	7.12e-1

*Term combination p=0.515

Table S13. The enrichment analysis result of SLPR on renal cell carcinoma dataset. The annotations are the same as in Table S10.

GO ID	Description	Rank	p-values
**GO: 0072073**	kidney epithelium development	1	2.26e-11
GO: 0007588	excretion	2	1.21e-10
**GO: 0001822**	kidney development	3	1.95e-9
**GO: 0048878**	chemical homeostasis	7	1.48e-7
**GO: 0009888**	tissue development	8	1.55e-7
**GO: 0003008**	system process	31	1.67e-5
**GO: 0044763**	cellular process	44	3.91e-5
**GO: 0043436**	oxoacid metabolic process	122	7.27e-4
**GO: 0006082**	organic acid metabolic process	125	8.53e-4
**GO: 0043170**	macromolecule metabolic process	2430	9.75e-1
**GO: 0046483**	heterocycle metabolic process	2446	9.99e-1

*Term combination p=4.752e-2

Reference

1 Barrett, T. & Edgar, R. Gene expression omnibus: microarray data storage, submission, retrieval, and analysis. *Methods Enzymol* **411**, 352-369, doi:10.1016/S0076-6879(06)11019-8 (2006).

2 Spira, A. *et al.* Airway epithelial gene expression in the diagnostic evaluation of smokers with suspect lung cancer. *Nat Med* **13**, 361-366, doi:10.1038/nm1556 (2007).

3 Noble, C. L. *et al.* Regional variation in gene expression in the healthy colon is dysregulated in ulcerative colitis. *Gut* **57**, 1398-1405, doi:10.1136/gut.2008.148395 (2008).

4 Scotto, L. *et al.* Identification of copy number gain and overexpressed genes on chromosome arm 20q by an integrative genomic approach in cervical cancer: potential role in progression. *Genes Chromosomes Cancer* **47**, 755-765, doi:10.1002/gcc.20577 (2008).

5 Pena-Llopis, S. *et al.* BAP1 loss defines a new class of renal cell carcinoma. *Nat Genet* **44**, 751-759, doi:10.1038/ng.2323 (2012).

6 Binns, D. *et al.* QuickGO: a web-based tool for Gene Ontology searching. *Bioinformatics* **25**, 3045-3046, doi:10.1093/bioinformatics/btp536 (2009).
